# Profile Groups Based on Lifestyle and Differences in Mental Health and Cognition

**DOI:** 10.1093/geroni/igaa057.1301

**Published:** 2020-12-16

**Authors:** Joseph Kim, Kyuree Kim

**Affiliations:** Iowa State University, Ames, Iowa, United States

## Abstract

The purpose of this study was to identify the profiles of older adults according to lifestyle. Data for the study were from the 2017 Consumption and Activities Mail Survey (CAMS). CAMS 2017 is a questionnaire mailed to a sub-sample of respondents from the Health and Retirement Study. Participants were limited to older adults 65 and older, and the final sample consisted of 1136 older adults. The sample included 443 men and 693 women. Caucasians comprised 82.0% of the participants. Lifestyle was measured through items assessing the amount of time spent on activities. Due to high skewness, the items were dichotomized, 0=no time spent on activity and 1=time spent on the activity. Latent class analysis (LCA) was performed to identify groups based on lifestyle. LCA is a person-centered approach for identifying unobserved subgroups based on similarity in responses to items. Three lifestyle groups were identified. Group 1 was “Outgoing” with 471 individuals. Group 2 was “Adequate” with 229 individuals. Group 3 was “Inactive” with 436 individuals. An ANOVA was then conducted to assess mean differences in self-rated health, cognition, depressive symptoms, and loneliness for the three lifestyle groups. The “Outgoing” and “Adequate” groups had significantly higher scores on self-rated health and cognition, and in addition, significantly lower scores on depressive symptoms and loneliness compared to the “Inactive” group. No significant differences were observed between the “Outgoing” and “Adequate” groups. An implication from this study is the importance of maintaining an active lifestyle in later life for better mental health and cognition.

